# Post-thyroidectomy ultrasonography versus thyroglobulin as a surveillance tool for locoregional recurrence in patients with differentiated thyroid carcinoma: A single centre 10-year study

**DOI:** 10.3389/fendo.2025.1594721

**Published:** 2025-11-10

**Authors:** Abhishek Mahajan, Vineeth Kurki, Pranjal Rai, Nilesh Sable, Ujjwal Agarwal, Richa Vaish, Nivedita Chakrabarty, Shreya Shukla, Anil D’Cruz, Prathamesh Pai, Kumar Prabhash, Vanita Noronha, Vijay Patil, Nandini Menon, Sarbani Ghosh Laskar, Asawari Patil, Munita Bal, Swapnil Rane, Neha Mittal, Pankaj Chaturvedi

**Affiliations:** 1 Department of Imaging, The Clatterbridge Cancer Centre National Health Service (NHS) Foundation Trust, Liverpool, United Kingdom; 2 Department of Radiodiagnosis and Imaging, Tata Memorial Hospital, Homi Bhabha National Institute, Mumbai, Maharashtra, India; 3 Department of Head and Neck Surgery, Tata Memorial Hospital, Homi Bhabha National Institute, Mumbai, Maharashtra, India; 4 Department of Medical Oncology, Tata Memorial Hospital, Homi Bhabha National Institute, Mumbai, Maharashtra, India; 5 Department of Radiation Oncology, Tata Memorial Hospital, Homi Bhabha National Institute, Mumbai, Maharashtra, India; 6 Department of Pathology, Tata Memorial Hospital, Homi Bhabha National Institute, Mumbai, Maharashtra, India

**Keywords:** thyroid cancer, differentiated thyroid carcinoma, ultrasonography, thyroidectomy, thyroglobulin, surveillance

## Abstract

**Background:**

Differentiated thyroid cancer (DTC; including papillary and follicular thyroid cancers) has favourable survival outcomes, with related mortality below 10%. However, 20–30% of patients experience recurrence. Surveillance primarily relies on neck ultrasonography (US) and serum thyroglobulin (Tg) assessment.

**Purpose:**

This study evaluated the diagnostic performance of neck US in detecting locoregional recurrence following total thyroidectomy and compared its effectiveness with serum Tg.

**Materials and methods:**

This retrospective, single-centre study analysed 941 DTC patients who underwent total thyroidectomy and neck US between 2009 and 2019. Suspicious US findings were correlated with serum Tg levels and anti-thyroglobulin antibody status. Disease persistence (<6 months)/recurrence (>6 months) was confirmed via fine-needle aspiration cytology/biopsy, iodine scintigraphy, CT, or PET-CT. Patients without US-detected lesions were assessed clinically, biochemically, and via follow-up US.

**Results:**

Neck US had a sensitivity of 98.9%, specificity of 63.1%, positive predictive value (PPV) of 50.7%, negative predictive value (NPV) of 99.3%, and an accuracy of 73.01%. Serum Tg (cutoff 1.8 ng/ml derived from receiver operating characteristic analysis) had a sensitivity of 69.2%, specificity of 91.8%, PPV of 61.4%, NPV of 94.1%, and an accuracy of 88.28%. Among 149 patients with US-detected lesions and Tg <1.8 ng/ml, 22 (14.8%) had locoregional recurrence. Five of 43 patients with Tg <0.1 ng/ml had confirmed recurrence. Among lymph nodes ≤6 mm in short-axis diameter with an indistinct fatty hilum, 69.6% were benign. Persistence was detected in 38.5% of patients within six months post-treatment, whereas most true recurrences (61.5%) manifested after six months.

**Conclusion:**

Neck US is highly sensitive but moderately specific for detecting locoregional recurrence post-thyroidectomy, complementing Tg. Study limitations include its retrospective design, single-centre setting, and lack of inter-observer variability assessment. A risk-adapted multimodal surveillance strategy with 6-monthly US for two years is recommended.

## Introduction

Thyroid cancer is the most prevalent endocrine malignancy, with its incidence steadily increasing over the past two decades due to factors such as improved healthcare accessibility and widespread thyroid imaging utilization (1, 2). Papillary thyroid carcinoma (PTC) accounts for 75–85% of cases and, together with follicular thyroid carcinoma (FTC), is classified as differentiated thyroid carcinoma (DTC) (3). The standard treatment for thyroid cancer includes near-total or total thyroidectomy, with neck dissection performed in cases of nodal metastases detected via preoperative imaging. Patients with distant metastases, commonly in the lungs or bones, undergo total thyroidectomy followed by radioactive iodine (RAI) uptake scanning and I-131 ablation therapy for remnant ablation or targeted metastatic treatment (4).

Most patients who receive appropriate treatment exhibit favourable survival outcomes, with cancer-related mortality rates below 10%. However, recurrence occurs in approximately 20–30% of cases, predominantly within the neck, either in the thyroid bed or lymph nodes, typically within the first decade post-treatment (5, 6). Regular follow-up involves physical examinations, ultrasound (US) assessments, and periodic serum thyroglobulin (Tg) measurements. Additional imaging modalities such as computed tomography (CT), magnetic resonance imaging (MRI), 18-fluorodeoxyglucose positron emission tomography (^18^FDG-PET), or I-131 scanning may be employed based on clinical indications to detect residual disease or recurrence at an early stage. High-resolution neck US is considered the primary surveillance tool for detecting persistent or recurrent disease. The American Thyroid Association (ATA) recommends serum Tg measurement with anti-Tg antibodies (TgAb) at 6- to 12-month intervals post-surgery and/or RAI therapy, alongside periodic neck US evaluations (7).

Serum thyroglobulin (Tg) remains a cornerstone biochemical marker for post-thyroidectomy monitoring, yet its utility is limited in certain scenarios. Approximately one-fourth of DTC patients develop anti-thyroglobulin antibodies that interfere with assay results, and Tg levels may remain low despite residual or recurrent disease, particularly in non–radioiodine-avid or non-ablated patients (8–10). Although ultrasensitive Tg assays (functional sensitivity <0.1 ng/mL) have improved detection thresholds and reduced need for TSH stimulation, their standalone specificity remains modest, warranting integration with structural imaging modalities such as US for accurate disease surveillance (11, 12). Consequently, neck US, being non-invasive, widely available, radiation-free, and highly sensitive, remains the preferred modality for detecting abnormal lymph nodes or masses in the thyroid bed. The integration of colour Doppler enhances the assessment of thyroid bed nodules for malignant characteristics, though confirmation via fine-needle aspiration cytology is warranted (10, 13).

Despite its utility, ultrasonography has limitations, including operator dependency and the potential for false-positive findings leading to unnecessary additional testing. Emerging applications of artificial intelligence (AI) and machine learning (ML) are increasingly being explored to address these limitations by reducing operator dependency and enhancing diagnostic specificity. Deep learning models trained on large annotated ultrasound datasets can automatically detect and characterize key sonographic features, thus showing promise in improving reproducibility, streamlining workflow, and mitigating reader variability in thyroid imaging (14).

The ATA acknowledges that some residual disease often remains post-thyroidectomy and emphasizes the role of neck US in identifying or excluding neoplastic disease, even in patients with undetectable serum Tg levels (15). However, studies evaluating US efficacy in patients with undetectable Tg levels report variable findings, with one recent study identifying recurrence in only 1 out of 170 such patients (16). Furthermore, certain studies highlight a high rate of false-positive abnormalities on neck US, leading to excessive testing without detecting clinically significant disease (16). An inherent limitation of local US is also its inability to adequately assess hematogenous dissemination to distant sites, particularly in certain subtypes of thyroid carcinoma such as follicular carcinoma.

The primary outcomes of this study were to determine the incidence of abnormal findings on neck US in post-total thyroidectomy patients with DTC and to evaluate their correlation with persistent or recurrent locoregional disease. Secondary outcomes included establishing the relationship between Tg levels and locoregional disease persistence or recurrence in DTC patients.

## Materials and methodology

This single-centre retrospective analytical study included patients who underwent neck US examinations at our institution over a 10-year period (1^st^ January 2009 to 31^st^ December 2019). The study population comprised patients who met specific inclusion and exclusion criteria based on clinical history, imaging records, and surgical and biochemical data.

Inclusion criteria required patients to have undergone neck US within the designated study period, with corresponding reports accessible in the radiology information system. Eligible patients had also undergone total thyroidectomy, with or without neck dissection or adjuvant radioiodine therapy, or had undergone revision surgery for suspected or confirmed recurrence, with or without additional radioiodine therapy. Exclusion criteria included a diagnosis of thyroid neoplasms other than DTC, treatment limited to lobectomy alone, or an indeterminate status regarding disease persistence or recurrence.

### Image analysis (Ultrasonography review)

Neck US examinations were performed using high-frequency linear transducers (7–15 MHz) on one of two ultrasound systems: LOGIQ E9 and LOGIQ E10 (GE Healthcare, Waukesha, WI, USA) during the ten-year study period. Across hardware upgrades, scanning presets were standardized to a departmental protocol encompassing the thyroid bed and cervical nodal levels I–VI, with uniform gain, depth, and Doppler sensitivity settings. Colour and power Doppler were employed for vascularity assessment. All examinations were conducted by fellowship-trained head-and-neck radiologists with a minimum of three years of post-training experience in thyroid imaging. Operators were blinded to contemporaneous Tg values and prior imaging results at the time of image acquisition and interpretation to minimize expectation bias.

US reports of all eligible patients were reviewed for the presence of abnormal features, including thyroid bed nodules, nodal masses, and heterogeneous lesions. The characteristics of these lesions were extracted from the radiology reports, including echogenicity (hypoechoic, hyperechoic, or isoechoic), vascularity (increased vascularity on Doppler imaging), calcifications (microcalcifications or coarse calcifications), lesion size (measured in millimetres), lymph node location (levels as per standard classification), and the short-axis diameter of the largest lymph node.

### Persistence and recurrence assessment

Diagnosis of persistence or recurrence was confirmed through fine-needle aspiration (FNA) cytology or biopsy in most cases. In keeping with standard definitions, disease identified within six months of surgery was categorized as persistence rather than recurrence. Additionally, patients with elevated serum Tg levels and/or high clinical suspicion (new/progressive symptoms: neck swelling, palpable nodes, dysphagia, hoarseness, dyspnoea, or rising TgAb titres despite undetectable serum Tg) underwent further diagnostic imaging, including iodine scintigraphy, CT, or ^18^FDG-PET-CT. Patients without identifiable lesions on US were assessed based on clinical and biochemical markers to confirm disease absence. Those with structural lesions who did not undergo FNA cytology or advanced imaging were monitored through serial US, and lesion stability was used to determine disease-free status.

Additional clinical and imaging data were extracted from electronic medical records to identify factors associated with recurrence. The following parameters were noted: age at the time of surgery, histopathological type of thyroid carcinoma, time interval between surgery and first postoperative ultrasonography, number of follow-up US examinations, T and N staging (extracted from preoperative imaging and postoperative histopathology reports, when available), and serum Tg levels (quantified using chemiluminescent immunometric assay; functional sensitivity of assay: 0.2 ng/mL and limit of detection: 0.1 ng/mL) and TgAb status (in patients with DTC, when available) to correlate with persistence or recurrence. It should be noted that all Tg values in this series represent basal, non–TSH-stimulated measurements. Missing biochemical data were excluded from comparative analyses on a pairwise deletion basis without imputation, as the missingness was judged to be random based on available metadata. All other clinical and imaging variables were complete for statistical analysis.

### Statistical analysis

Data were summarized using the median and interquartile range for continuous variables, while categorical variables were presented as frequencies and percentages. The normality of variables was assessed using the Shapiro-Wilk test. The primary objective was evaluated through diagnostic test performance, including sensitivity, specificity, positive predictive value (PPV), and negative predictive value (NPV) of US in detecting locoregional persistence or recurrence. Sensitivity and PPV were calculated for each US feature, and categorical variables in the locoregional persistence/recurrence group were compared with the non-recurrence group using the chi-square test or Fisher’s exact test. Continuous variables were analysed using the Mann-Whitney U test. Odds ratios (ORs) with corresponding 95% confidence intervals (CIs) were calculated to estimate the strength of association for each predictor. For correlation between Tg levels and disease persistence/recurrence in patients without TgAbs, a cut-off value was determined using the receiver operating characteristic (ROC) curve. Logistic regression analysis was conducted to evaluate the association between US findings and Tg levels. Data were analysed using IBM SPSS v25, and a p-value of <0.05 was considered statistically significant.

## Results

### Clinical and ultrasonographic features

**Figure 1:**
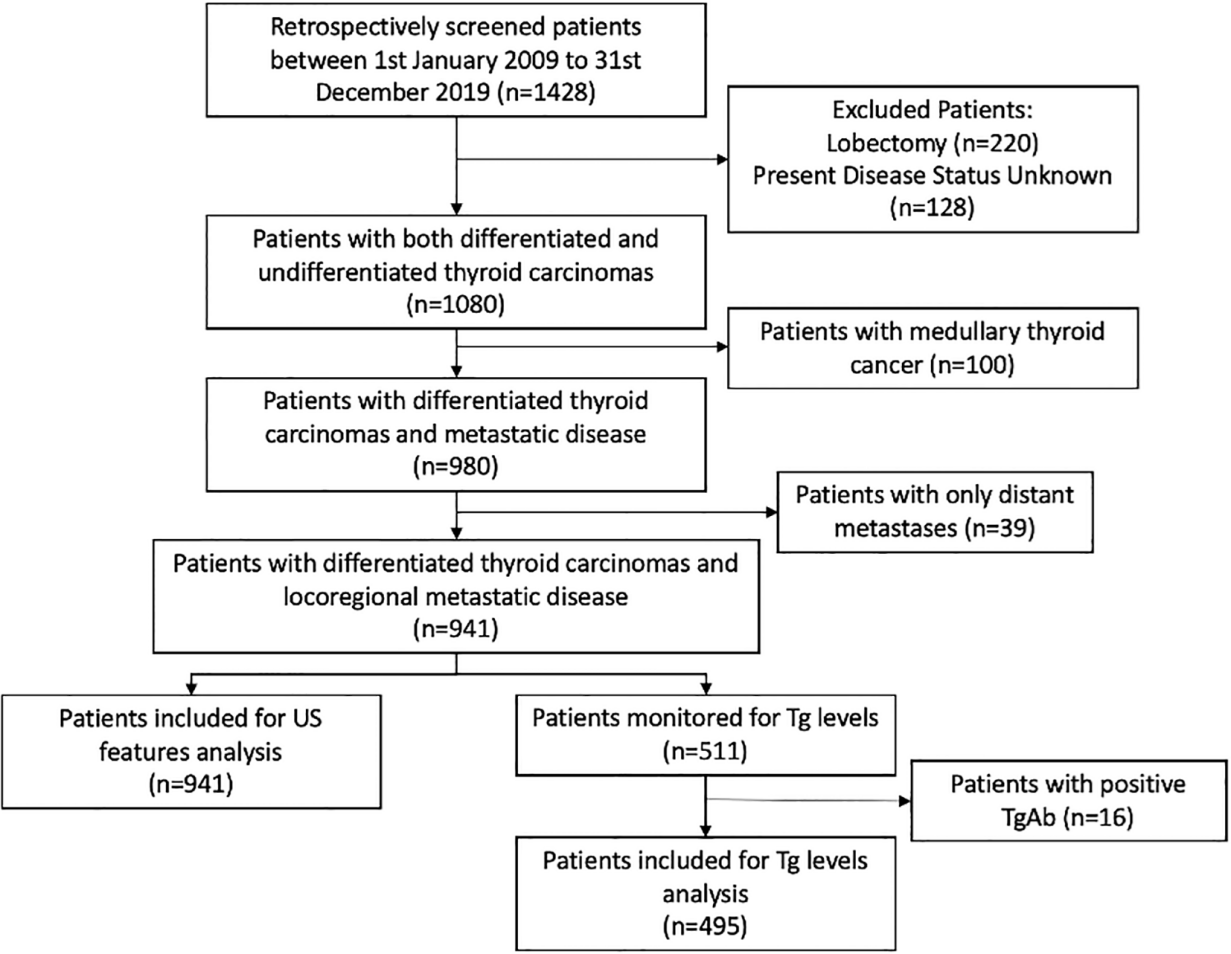
Flow chart demonstrating patient selection and inclusion.

Final sample size was 941 patients (**Figure 1**). Patient characteristics are summarized in **Table 1**, and US features are presented in **Table 2**. Patients were classified into two groups based on the presence or absence of disease. There was no statistically significant difference between the groups in terms of median age at surgery (42 [IQR 30–53] *vs* 39 [IQR 29–48] years; median difference = 3 years, 95% CI –0.8 to 7.9; p = 0.126), gender (OR: 1.30; 95% CI: 0.97 to 1.76; p = 0.08), histopathology (OR: 1.12; 95% CI 0.54 to 2.33; p = 0.765), or type of surgery (OR: 1.17; 95% CI: 0.84 to 1.61; p = 0.36). Patients who underwent RAI therapy post-surgery had a lower recurrence rate (OR: 0.75; 95% CI: 0.55 to 1.00; p = 0.038) (**Table 3**).

**Table 1:** Clinical characteristics of the study cohort.

Variables	Values	
Sample size (N)	941	
Median age (years) [IQR]	38 [28–49]	
	Frequency	%
Gender		
Male	316	33.6
Female	625	66.4
Histopathology		
Papillary	902	95.9
Follicular	39	4.1
Radioactive Iodine Therapy		
Yes	507	53.9
No	434	46.1
Type of surgery		
Total thyroidectomy	226	24
Total thyroidectomy and neck dissection	715	76
Median interval between surgery and first post operative USG [IQR]	8 months [2–22]	
Mean number of USGs	1.94	
Median interval between USGs [IQR]	10 months [6–14]	
Thyroglobulin levels available	511	
Anti-Thyroglobulin Antibody (TgAb) interference	16	

**Table 2:** Ultrasonographic features of detected lesions.

Sites in USG		Frequency	%
Lesions in Bed		122	13
Lesions in Bed with suspicious nodes		115	12.2
Only Nodes		312	33.2
Number of lesions			
Single		182	19.3
Multiple		297	31.6
Echogenicity in USG			
Hypoechoic	Lesion in bed	152	64.1
	Nodes	171	54.8
Hyperechoic	Lesion in bed	34	14.3
	Nodes	4	1.3
Heteroechoic	Lesion in bed	37	15.6
	Nodes	56	17.9
Microcalcifications			
Present	Bed	69	29.1
	Nodes	57	18.3
Absent		22	2.3
Vascularity			
Present	Bed	87	36.7
	Nodes	21	6.7
Absent		33	3.5
Fatty hilum	Disrupted	136	14.5
	Indistinct	95	10.1
	Maintained	7	0.7
Neck nodes compartment	Central	82	8.7
	Lateral	263	27.9
	Both	78	8.3

Median size of largest bed lesion (mm)[IQR] – 9 (7–13).

Median short axis diameter of largest node (mm)[IQR] – 7 (5–10).

**Table 3:** Clinical comparison between patients with and without disease recurrence.

Clinical Parameter	Persistence/recurrence – 261 (27.7%)	No persistence/recurrence – 680 (72.3%)	p Value [OR/median difference for age (95% CI)]
Median age (Years)[IQR]	42[30–53]	39[29–48]	0.126 [3 years (–0.8 to 7.9)]
Gender			
Male	99 (31.3)	217 (68.7)	0.08 [1.30 (0.97 to 1.76)]
Female	162 (25.9)	463 (74.1)	
Histopathology			
Papillary	251 (27.8)	651 (72.2)	0.765 [1.12 (0.54 to 2.33)]
Follicular	10 (25.6)	29 (74.4)	
Radio-active Iodine			
RAI ablation	128 (25.2)	379 (74.8)	0.038 [0.75 (0.55 to 1.00)]
No Ablation	133 (30.6)	301 (69.4)	
Type of surgery			
Total thyroidectomy	68 (30.1)	158 (69.9)	0.365 [1.17 (0.84 to 1.61)]
TT + nodal dissection	193 (27.0)	522 (73.0)	

**Figure 2:**
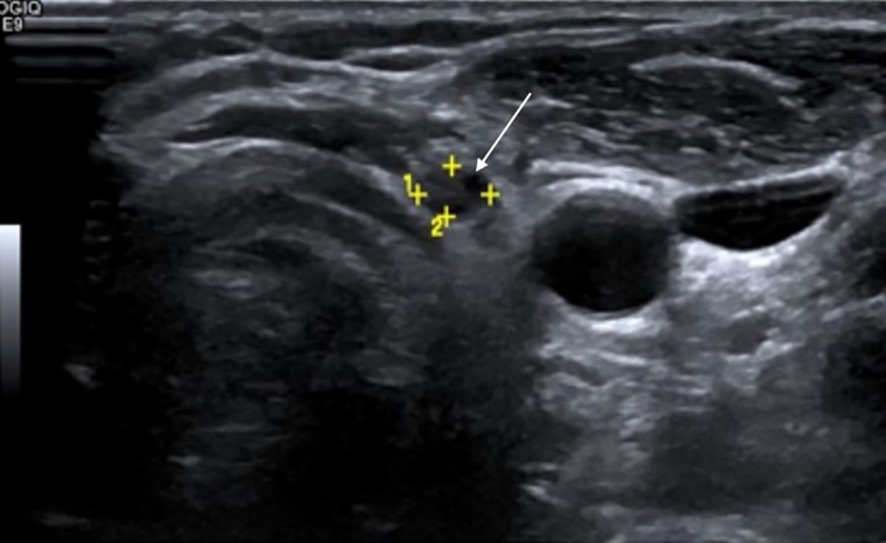
Surveillance greyscale ultrasound of the left thyroid bed in a 28-year-old female with differentiated thyroid carcinoma (DTC) post-total thyroidectomy shows a suspicious hypoechoic nodule with irregular margins (white solid arrow)—features suspicious for recurrence. Serum thyroglobulin (Tg) level was 0.9 ng/mL. Fine-needle aspiration cytology (FNAC) revealed clusters of atypical follicular cells, confirming recurrent disease.

**Figure 3:**
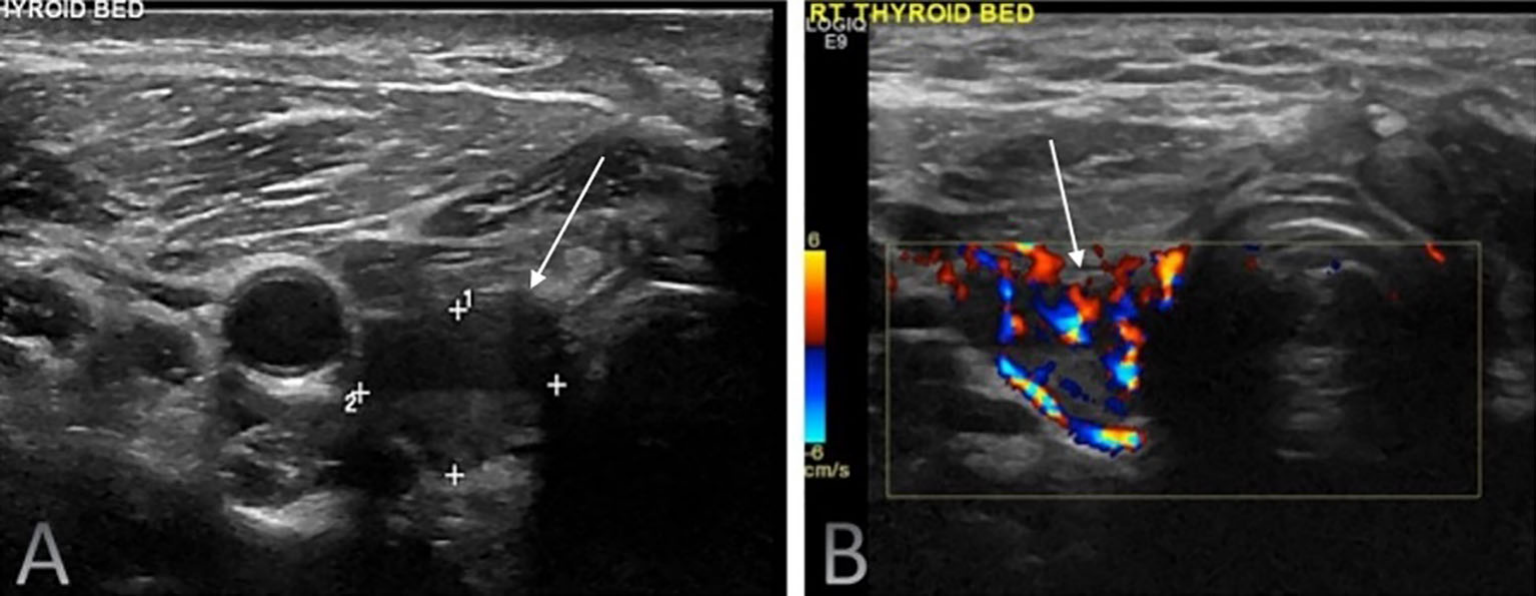
Routine surveillance duplex ultrasound of the right thyroid bed in a 41-year-old male with differentiated thyroid carcinoma (DTC) following total thyroidectomy and radioactive iodine therapy. The imaging demonstrated a highly suspicious solid hypoechoic nodule (white solid arrows) with loss of normal fascial planes on greyscale ultrasound **(A)** with internal vascularity seen on colour doppler **(B)**. The serum Tg level was 8.6 ng/mL. Fine-needle aspiration cytology confirmed recurrent papillary thyroid carcinoma.

US findings significantly associated with locoregional disease presence included hypoechoic or heteroechoic thyroid bed lesions with microcalcifications and vascularity (**Figures 2**, **3**), as well as heteroechoic nodes with abnormal vascularity and loss of fatty hilum. Patients with nodal involvement in both central and lateral neck compartments had a higher risk of disease. On bivariate analysis, the presence of abnormal vascularity in the thyroid bed was associated with a significantly higher risk of disease (OR: 4.4; 95% CI: 1.024 to 18.897, p=0.046). The presence of microcalcifications in the thyroid bed was also significantly associated with an increased risk of disease (OR: 7.36; 95% CI: 1.845 to 29.36, p=0.005).

### Accuracy of ultrasonography and serum Tg levels

Overall, US exhibited a sensitivity of 98.9%, specificity of 63.1%, positive predictive value (PPV) of 50.7%, and negative predictive value (NPV) of 99.3%, with an accuracy of 73.01%. Among 509 patients with US-detected abnormalities, 258 had confirmed locoregional disease, while 3 patients with negative US findings were later diagnosed with locoregional recurrence via PET-CT and confirmed by FNA cytology (**Supplementary Tables 1A, B**).

**Figure 4:**
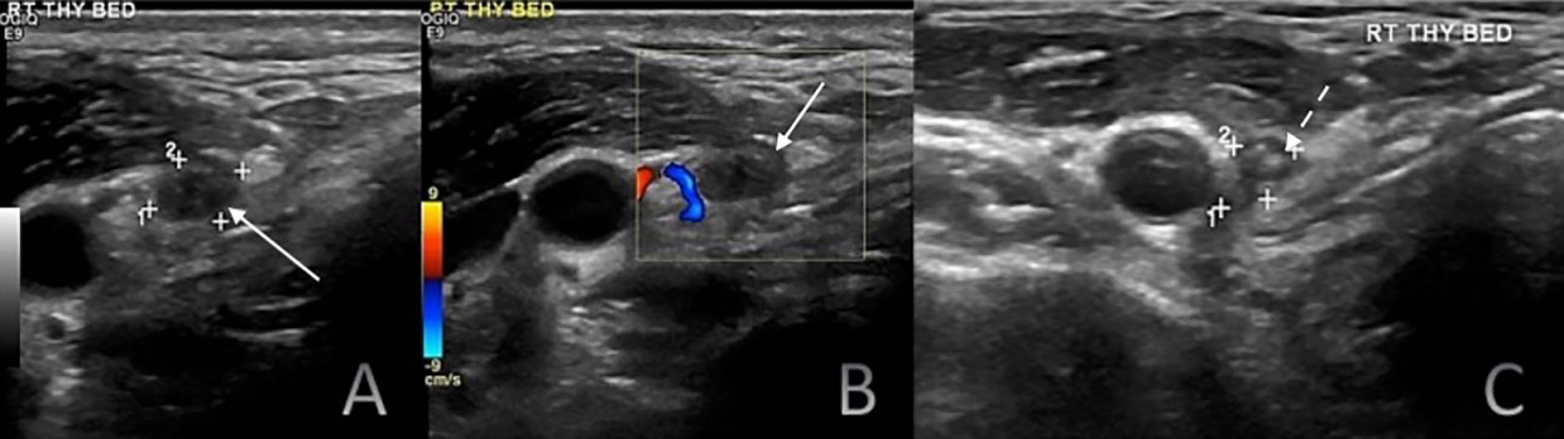
Six-month postoperative surveillance duplex ultrasound of the right thyroid bed demonstrating a hypoechoic nodule (white solid arrows) on greyscale ultrasound **(A)** without detectable internal vascularity on color doppler imaging **(B)**. The nodule contained few hyperechoic foci (C; white dashed arrows). Although the lesion appeared suspicious on greyscale imaging, the absence of vascularity and serum Tg level of 11.2 ng/mL suggested a benign process. Fine-needle aspiration cytology established the diagnosis of a foreign body granuloma.

Serum Tg (cutoff: 1.8 ng/ml in TgAb-negative patients) had a sensitivity of 69.2%, specificity of 91.8%, PPV of 61.36%, and NPV of 94.1%, with an accuracy of 88.28% (**Supplementary Tables 2A, B**). Among 34 patients with Tg levels >1.8 ng/ml but no structural disease, 8 had levels >10 ng/ml. In 3 of these patients, FNA of suspicious lesions confirmed post-operative changes (**Figure 4**) and reactive nodes. Two underwent PET-CT, which showed no locoregional disease, while 3 remained stable on follow-up US. Among these 34 patients, RAI was administered selectively, based on disease stage, risk stratification, and multidisciplinary tumour board recommendations with 9 patients not receiving RAI. Four patients had residual thyroid, and four underwent multiple surgeries but remained clinically disease-free. Area under curve for the ROC analyses was 0.83 (95% CI: 0.79-0.87).

Among 149 patients with Tg <1.8 ng/ml and no TgAbs, 22 (14.8%) had proven locoregional disease: 13 had nodal metastases, 6 had thyroid bed recurrence, and 3 had both. Among 43 patients with Tg <0.1 ng/ml, 5 (11.6%) had recurrence/persistence, including 4 with nodal metastases and 1 with thyroid bed recurrence diagnosed 9 years post-surgery, possibly due to dedifferentiation. The time from surgery to nodal metastasis detection ranged from 1 month to 3 years. Patients with Tg >1.8 ng/ml had a 13.86-fold higher risk of recurrence (95% CI: 7.64- 25.16; p < 0.001) than those with lower levels. Suspicious US findings conferred a 7.13-fold increased risk of recurrence compared to indeterminate or negative US findings (95% CI: 3.95-12.89; p < 0.001).

### Challenges in small cervical nodes

Among 112 patients with cervical nodes ≤6 mm in short-axis diameter and an indistinct or absent fatty hilum, 34 (30.4%) had nodal metastases, while 78 (69.6%) did not. In contrast, all 4 patients with a maintained fatty hilum had reactive nodes rather than metastatic disease.

### Timing of disease recurrence/persistence

Of all cases with disease detected during follow-up, 38.5% occurred within six months of surgery and were therefore classified as persistent disease, reflecting residual tumour or nodal metastases not eradicated at initial treatment. The remaining cases, detected beyond six months after a documented disease-free interval, were considered true recurrences (**Table 4**).

**Table 4:** Timeline of disease recurrence following surgery.

S.No.	Interval between surgery and diagnosis of persistence/recurrence*	Percentage of total patients with persistence/recurrence	Cumulative Percentage of total patients with persistence/recurrence
1	0–6 months	38.5	38.5
2	7 months to 1 year	16.5	55.0
3	1 year to 2 years	15.6	70.6
4	2 years to 5 years	18.1	88.7
5	5 years to 10 years	8.7	97.4

* Recurrence timing categorized as persistent (≤6 months) versus true recurrence (>6 months).

**Supplementary Figure 1** shows a forest plot for odds ratio for various parameters predicting recurrence in the present study.

## Discussion

Surveillance for recurrent or persistent disease in post-thyroidectomy patients is critical, and US and serum Tg estimation remain cornerstone modalities in the follow-up of patients with DTC. US is especially valuable in monitoring residual thyroid tissue, detecting locoregional recurrent disease, and evaluating neck nodal metastases (17, 18). In this study, we examined the diagnostic performance of US and serum Tg, assessed the role of small nodal metastases, and evaluated recurrence timing to optimize surveillance strategies.

Locoregional recurrence rates in thyroid cancer range from 9-30%, depending on initial tumour burden, surgical completeness, and adjuvant therapy. In our cohort, 261 of 941 patients (27.7%) developed recurrence, aligning with the findings of Luo et al., who reported a 12% recurrence rate (19). The vast majority of thyroid bed recurrences (97.4%) were diagnosed within the first decade post-surgery, with a median interval of 11 months, emphasizing the need for early and sustained surveillance. Although 2.6% of recurrences occurred beyond 10 years post-surgery (mean interval: 12.1 years), these late recurrences suggest the possibility of slow-growing clones or dedifferentiation over time.

Patient age at surgery did not significantly impact recurrence rates, consistent with studies by Luo et al. and Ryoo et al. (2, 19). Furthermore, although neck dissection at the time of thyroidectomy was associated with a lower recurrence rate, this finding was not statistically significant, mirroring prior reports from Luo et al. (19). Postoperative RAI therapy significantly reduced recurrence rates, consistent with Toniato et al. (20), reinforcing its role in reducing microscopic residual disease and improving long-term disease-free survival.

US demonstrated high sensitivity (98.9%) and NPV (99.3%) in detecting locoregional disease, confirming its role as a first-line imaging modality. However, its specificity (63.1%) was relatively low, likely due to false positives in patients with post-surgical changes or reactive nodes. These limitations are consistent with a prior study by Simeone et al. (21), who also reported high US sensitivity but reduced specificity in the post-operative setting.

Serum Tg was less sensitive than US but had higher specificity (91.8%). A Tg cut-off of 1.8 ng/ml was associated with an increased risk of recurrence (OR: 13.86). The cut-off of 1.8 ng/mL was derived from ROC analysis within our cohort to maximize sensitivity and specificity; however, it should be acknowledged that this relatively high threshold may inevitably miss low-level recurrences. Despite its strong predictive value, Tg alone was insufficient for ruling out disease, as 22 of 149 patients with Tg <1.8 ng/ml still had proven recurrence. Additionally, 11.6% of patients with undetectable Tg levels (<0.1 ng/ml) developed recurrence, consistent with prior studies by Pacini et al., who reported that even in patients with undetectable Tg, 1.2% had true recurrence (22). This highlights the limitations of Tg in detecting low-volume or structurally occult disease and reinforces the need for concurrent US evaluation.

In our study, Tg demonstrated higher overall diagnostic accuracy than US (88.3% *vs*. 73.0%), reflecting its strength as a biochemical marker of recurrence. Nonetheless, Tg alone may be insufficient, as 11.6% of patients with undetectable Tg (<0.1 ng/mL) still harboured proven disease. US remains indispensable as the primary structural detector, and when combined with basal Tg, both sensitivity and NPV can reach 100%, while also reducing the need for stimulation testing in nearly 80% of patients (12). This complementary role underscores that Tg provides greater overall accuracy, but US ensures detection of structurally occult locoregional disease.

Our results demonstrated that US findings, such as hypoechoic or heteroechoic thyroid bed lesions with microcalcifications and vascularity, were significantly associated with recurrent thyroid-bed disease. Similarly, heteroechoic nodes with abnormal vascularity and loss of fatty hilum were predictive of nodal metastases. The presence of nodal involvement in both central and lateral neck compartments conferred a higher risk of recurrence, underscoring the importance of systematic nodal evaluation during US surveillance. These findings align with those of Leboulleux et al., highlighting the prognostic value of nodal architecture and vascularity in identifying malignant nodes (23).

An important challenge identified in our study was the evaluation of small cervical nodes. Among 112 patients with nodal lesions ≤6 mm, 30.4% had metastatic disease, demonstrating that small node size does not preclude malignancy, particularly when additional suspicious features such as loss of fatty hilum, abnormal vascularity, or microcalcifications are present. While prior studies (such as one by Kwak et al.) suggested that small nodes with preserved fatty hilum are more likely reactive (24, 25), our findings emphasize that short-axis diameter alone should not be the sole determinant in ruling out malignancy, While FNAB is technically challenging in very small nodes, it may be warranted in nodes ≤6 mm that demonstrate such suspicious sonographic characteristics.

Regarding recurrence timing, 38.5% of recurrences were diagnosed within 6 months post-surgery. Furthermore, 70.6% of recurrences occurred within the first 2 years, aligning with Ryoo et al., who suggested that early recurrences reflect incomplete initial treatment or residual microscopic disease (2). Our findings support US surveillance every 6 months for the first 2 years post-surgery for patients with any of the following: high-risk US features; Tg ≥1.8 ng/mL or rising Tg/TgAb; suspicious/indeterminate US findings; residual thyroid or no RAI; or multi-compartment nodal disease (central + lateral). For low-risk patients with undetectable basal Tg (<0.1 ng/mL) and a negative US at 6–12 months, de-escalate to annual US, then every 12–24 months thereafter. In TgAb-positive patients with negative imaging, perform US every 6–12 months until antibodies decline.

While our study reinforces the central role of US in post-thyroidectomy surveillance, it also highlights challenges of false-positive results, which may provoke patient anxiety, lead to unnecessary biopsies, and contribute to increased healthcare costs (25, 26). These limitations highlight the need for balanced surveillance strategies that minimize overtreatment without compromising early detection. Emerging AI and ML-based tools hold promise to reduce operator dependency and improve the specificity of US interpretation (14). Future prospective, multicentre trials incorporating AI-assisted US workflows (along with liquid biopsy assays), could provide an integrated, non-invasive framework for personalized recurrence monitoring.

Recent literature strongly emphasizes the complementary role of neck US and serum Tg for DTC surveillance, as each modality offsets the other’s limitations. Tg testing serves as an efficient high-sensitivity screen: if Tg remains undetectable (and anti-Tg antibodies are absent), the likelihood of active structural disease is exceedingly low. After total thyroidectomy and RAI, surveillance US can be safely deferred in patients with low or undetectable Tg levels, since virtually no clinically significant recurrences occurred in Tg-negative, antibody-negative patients. In those patients, neck US is more likely to yield false-positive nodules than true cancer. On the other hand, when Tg is elevated or rising, ultrasound plays a crucial role in localizing the recurrence (27–29).

The strengths of this study include its large cohort and detailed ultrasonographic analysis of recurrent disease patterns. However, several limitations should be acknowledged. The retrospective, single-centre design may introduce inherent selection bias and limit generalizability, as only patients with available follow-up and complete biochemical and imaging data were included. US hardware evolved over the 10-year study period, introducing variability in doppler-based vascularity assessment. Despite protocol standardization, neither inter-device calibration nor inter-observer agreement testing was performed which could affect reproducibility. Follow-up intervals were inconsistent, reflecting real-world practice but limiting uniform comparisons. ATA recurrence risk stratification (low, intermediate, high) was not available for all patients due to the retrospective and imaging-focused design of this study, which may limit correlation between baseline risk and recurrence outcomes. Another limitation is that while near-total thyroidectomy was included in the cohort, the average volume of residual thyroid tissue in these cases was not quantified, which could potentially influence Tg interpretation. TSH levels were also not consistently measured at the time of Tg assessment, which may influence Tg interpretation. Tg measurements were derived from a heterogeneous cohort including both RAI-treated and non-RAI patients, which may confound the analysis of Tg’s diagnostic accuracy. We also specifically evaluated the role of US in detecting locoregional recurrence; its utility does not extend to assessing hematogenous dissemination to distant sites.

Despite these limitations, our findings reinforce the pivotal role of US and serum Tg in the surveillance of post-thyroidectomy patients, emphasizing the need for a risk-adapted, multimodal follow-up strategy. Future studies incorporating advanced imaging techniques and molecular markers may further refine recurrence detection and risk stratification, ultimately improving long-term patient outcomes.

## Conclusion

Neck US and serum Tg together provide a robust, generalizable framework for detecting recurrence after thyroidectomy. Broader implementation of this combined approach could standardize follow-up care across centres. Future research should focus on refining risk-adapted surveillance algorithms incorporating molecular markers and AI-assisted US to enhance early recurrence detection and optimize long-term management of thyroid cancer patients.

## Data Availability

The raw data supporting the conclusions of this article will be made available by the authors, without undue reservation.
