# Supplementary material for: Post-thyroidectomy ultrasonography versus thyroglobulin as a surveillance tool for locoregional recurrence in patients with differentiated thyroid carcinoma: A single centre 10-year study
Source: Front Endocrinol (Lausanne). 2025 Nov 10;16:1594721. doi: 10.3389/fendo.2025.1594721 (PMC12640809; doi:10.3389/fendo.2025.1594721)
Supplement: Supplementary file 1 [file Image1.pdf]

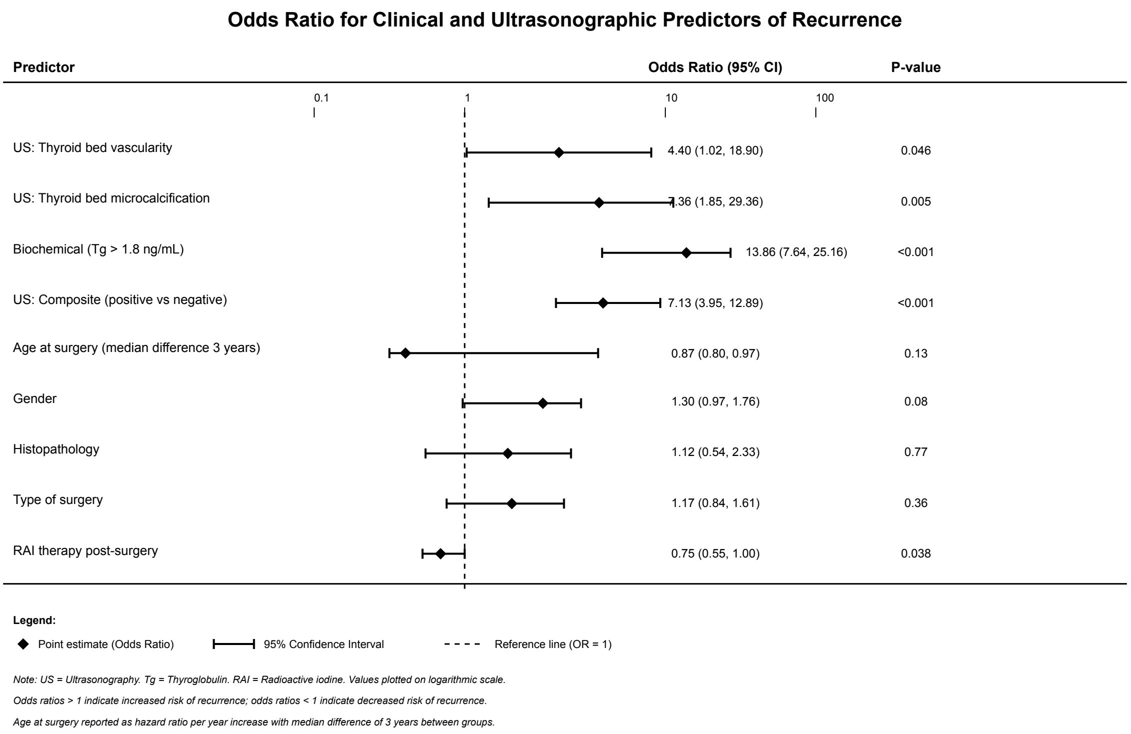

Supplementary Figure 1: Forest plot demonstrating odds ratios (ORs) with 95% confidence intervals for clinical and ultrasonographic predictors of recurrence in differentiated thyroid carcinoma.
