# Supplementary material for: Post-thyroidectomy ultrasonography versus thyroglobulin as a surveillance tool for locoregional recurrence in patients with differentiated thyroid carcinoma: A single centre 10-year study
Source: Front Endocrinol (Lausanne). 2025 Nov 10;16:1594721. doi: 10.3389/fendo.2025.1594721 (PMC12640809; doi:10.3389/fendo.2025.1594721)
Supplement: Supplementary file 2 [file Table1.docx]

**Supplementary Tables:**

**Supplementary Table 1A:** Cross-tabulation of ultrasound findings against reference-confirmed recurrence status. “Positive” denotes sonographic features suspicious for recurrence (e.g., hypoechoic nodules, irregular margins, microcalcifications, or abnormal vascularity). “Negative” denotes absence of such findings.

|  | | **Recurrence** | | **Total** |
| --- | --- | --- | --- | --- |
|  |  | **Yes** | **No** |  |
| **Ultrasound Report** | **Positive** | 258 | 251 | **509** |
|  | **Negative** | 3 | 429 | **432** |
| **Total** | | **261** | **680** | **941** |

**Supplementary Table 1B:** Diagnostic performance (Sensitivity, specificity, positive predictive value (PPV), negative predictive value (NPV), and overall accuracy) of ultrasound in detecting recurrence in differentiated thyroid carcinomas.

| **Metric** | **Value** |
| --- | --- |
| Sensitivity | 98.90% |
| Specificity | 63.10% |
| PPV | 50.70% |
| NPV | 99.30% |
| Accuracy | 73.01% |

**Supplementary Table 2A:** Cross-tabulation of Thyroglobulin (Tg) levels using a cutoff of 1.8ng/mL against reference-confirmed recurrence status.

|  |  | **Recurrence** | | **Total** |
| --- | --- | --- | --- | --- |
|  |  | **No** | **Yes** |  |
| **Tg level (ng/mL)** | **< 1.8** | 383 | 24 | **407** |
|  | **≥ 1.8** | 34 | 54 | **88** |
| **Total** | | **417** | **78** | **495** |

**Supplementary Table 2B:** Diagnostic performance (Sensitivity, specificity, positive predictive value (PPV), negative predictive value (NPV), and overall accuracy) of Tg level (≥ 1.8ng/mL) in detecting recurrence in differentiated thyroid carcinomas.

| **Metric** | **Value** |
| --- | --- |
| Sensitivity | 69.2% |
| Specificity | 91.8% |
| PPV | 61.4% |
| NPV | 94.1% |
| Accuracy | 88.3% |
